# Environmental context of phenotypic plasticity in flowering time in sorghum and rice

**DOI:** 10.1093/jxb/erad398

**Published:** 2023-10-11

**Authors:** Tingting Guo, Jialu Wei, Xianran Li, Jianming Yu

**Affiliations:** Hubei Hongshan Laboratory, Wuhan, Hubei, China; College of Plant Science and Technology, Huazhong Agricultural University, Wuhan, Hubei, China; Department of Agronomy, Iowa State University, Ames, IA, USA; USDA, Agricultural Research Service, Wheat Health, Genetics, and Quality Research Unit, Pullman, WA, USA; Department of Agronomy, Iowa State University, Ames, IA, USA; CSIRO Agriculture and Food, Australia

**Keywords:** Climate change, environmental variability, genomic prediction, genotype by environment interaction, phenotypic plasticity, plant–environment interactions, reaction norm

## Abstract

Phenotypic plasticity is an important topic in biology and evolution. However, how to generate broadly applicable insights from individual studies remains a challenge. Here, with flowering time observed from a large geographical region for sorghum and rice genetic populations, we examine the consistency of parameter estimation for reaction norms of genotypes across different subsets of environments and searched for potential strategies to inform the study design. Both sample size and environmental mean range of the subset affected the consistency. The subset with either a large range of environmental mean or a large sample size resulted in genetic parameters consistent with the overall pattern. Furthermore, high accuracy through genomic prediction was obtained for reaction norm parameters of untested genotypes using models built from tested genotypes under the subsets of environments with either a large range or a large sample size. With 1428 and 1674 simulated settings, our analyses suggested that the distribution of environmental index values of a site should be considered in designing experiments. Overall, we showed that environmental context was critical, and considerations should be given to better cover the intended range of the environmental variable. Our findings have implications for the genetic architecture of complex traits, plant–environment interaction, and climate adaptation.

## Teaser

Using flowering time from two genetic populations of sorghum and rice we demonstrate that both environment sample size and environmental mean range influence parameter estimation of phenotypic plasticity from different subsets of environments.

## Introduction

Global climate change has had observable effects on our planet, and the situation will worsen in the decades to come (Power *et al.*, 2021). Studying how plants respond to environmental changes, or phenotypic plasticity, not only can reveal the mechanisms of gene–environment interplay but also can allow an in-depth assessment of genetic diversity in the face of climate change. Previous phenotypic plasticity studies have revealed the patterns of genotypes responding to environmental changes (Kusmec *et al.*, 2017; Li *et al.*, 2018, 2021; Guo *et al.*, 2020; Liu *et al.*, 2021). However, the patterns detected in empirical studies will remain valid for new environments only if the major environmental variables are dominant and their values are within a certain range of biological relevance (Bonamour *et al.*, 2019). Therefore, further examination of how to sample the environments to study phenotypic plasticity is needed for gaining a better understanding of the environmental context of phenotypic variation, predicting the responses of organisms to climate change, and developing strategies to improve crop resilience to environmental stresses.

Common garden experiments (CGEs) are widely used in evolution and ecology to study phenotypic plasticity and the genetic basis of local adaptation (de Villemereuil *et al.*, 2016). Similarly, conducting multi-environment trials (METs) is an established approach in agriculture to quantify environmental variability and breed varieties targeting different regions (Piepho, 1998; Yan *et al.*, 2001; Crossa *et al.*, 2010; Malosetti *et al.*, 2013). Although the combination of sites and seasons for CGEs and METs are generally considered to be representative samples of a large geographical range and time and future climatic conditions in the target populations of environments, it might become ineffective under climate change, and the site selection needs to be periodically revisited (Cooper *et al.*, 2021). The design of CGEs and METs has gained interest recently with the objectives of selecting representative sites to cover the intended area and characterizing genotypes for their adaptability across the target population of environments. Various modeling techniques have shown their usefulness to maximize the accuracy of genotypic mean estimation (González-Barrios *et al.*, 2019; Prus and Piepho, 2021). On the other hand, there is limited research on the study design of phenotypic plasticity where the focus is to extract any pattern as a continuous environmental gradient from the set of discrete, tested environments.

Studies in genotype by environment interaction (G×E) and phenotypic plasticity have made progress in revealing the biological mechanisms of plant response to environmental variation (Wu and Lin, 2006; Des Marais *et al.*, 2012; Sasaki *et al.*, 2015; Mu *et al.*, 2022) and predicting trait performance in new environments (Jarquín *et al.*, 2014; Resende *et al.*, 2021; Li *et al.*, 2022). Because empirical studies generally involved a limited number of environments due to resource constraints (Clausen *et al.*, 1948; Ungerer *et al.*, 2003; Des Marais *et al.*, 2012; Sasaki *et al.*, 2015), it is not always feasible to extend the resulting findings to a larger context (Lowry *et al.*, 2019). Similarly, the developed prediction models need to be updated periodically to maintain their generalizability and to extend the prediction capacity to future environments at large.

**Fig. 1.**
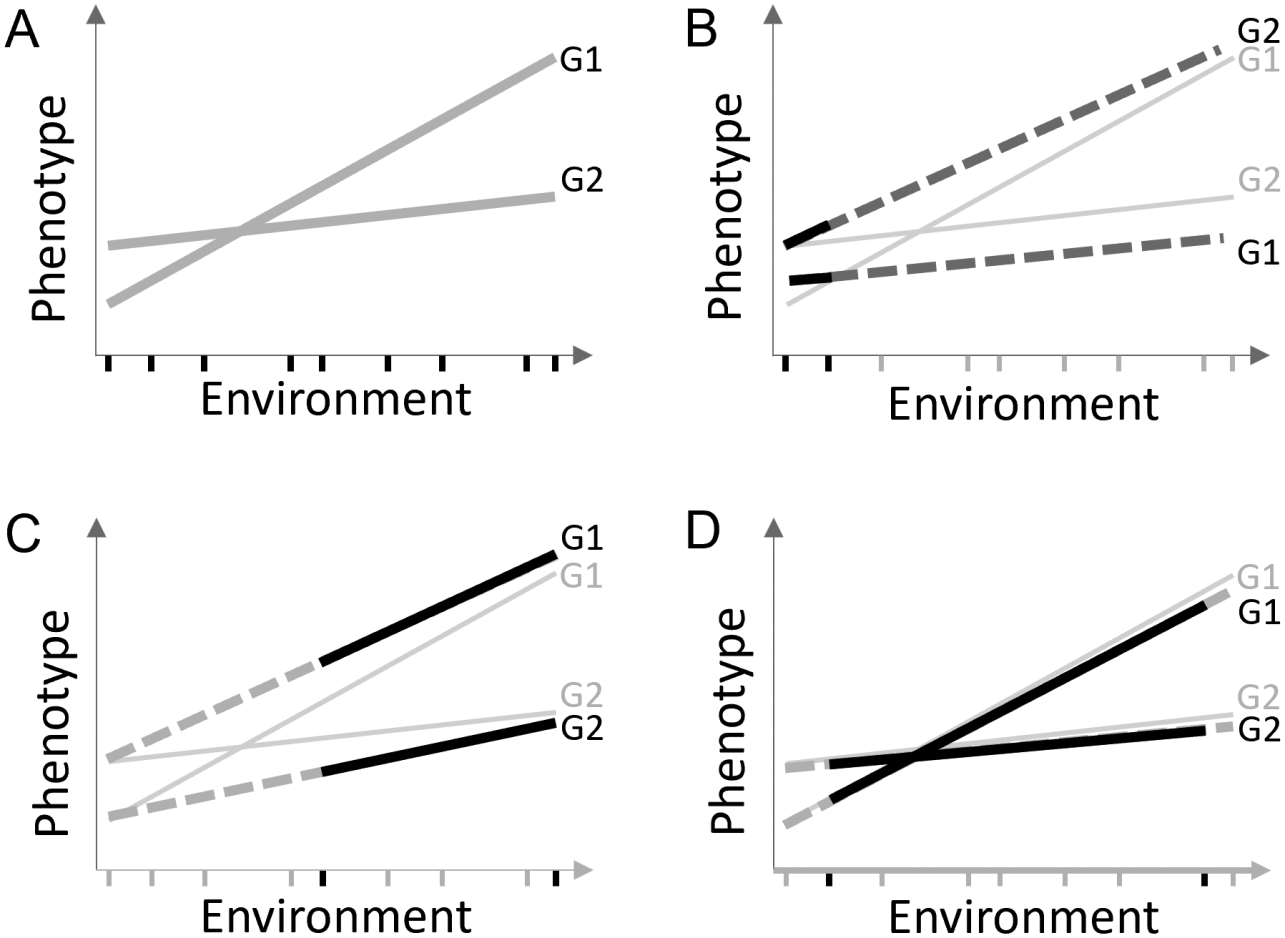
A schematic diagram of different scenarios of reaction norms. (A) Reaction norms of two genotypes (G1 and G2) across the entire range of environments. (B) Reaction norms obtained from a small range of environments. (C) Reaction norms obtained from a medium range of environments. (D) Reaction norms obtained from a large range of environments. In (B–D), bold lines indicate the obtained reaction norm within the studied range; dashed lines indicate the extrapolated sections, which can deviate in (B) and (C) from (A); light gray lines indicate the reaction norm across the entire range. In (A–D), bold ticks indicate environments used to obtain the reaction norms, and gray ticks indicate environments not used.

Experiments that evaluate genetic populations at a broad geographical region offer unique opportunities for examining the influence of variability of tested environments on the pattern of phenotypic plasticity (Fig. 1; Supplementary Fig. S1). Using flowering time from two genetic populations, sorghum [*Sorghum bicolor* (L.) Moench] and rice (*Oryza sativa* L.), we examined the consistency in parameter estimation for reaction norms of genotypes by altering environment sample size and environmental mean range. This goes beyond the earlier studies (Li *et al.*, 2018; Guo *et al.*, 2020), where the focus was to identify patterns of the whole set of environments. Next, we predicted the reaction norm parameters for untested genotypes with genome-wide markers and models developed from tested genotypes. Furthermore, by leveraging historical weather data and assuming a constant environmental index, we conducted simulations to reveal the environmental variability of different performance testing sites, providing information to prioritize sites with larger environmental variability. Accurate, cost-effective detection of the global pattern across a large geographic range in phenotypic plasticity studies provides information on how to design genetics research related to climate change adaptation and crop improvement.

## Materials and methods

### Genetic population, phenotyping, and genotyping

Two genetic populations were utilized as study materials. The sorghum population with 237 recombinant inbred lines (RILs) from a cross between Tx430 and B898012 was evaluated in nine environments. Within each environment, a randomized complete block design with two replications was used. The nine environments were denoted as PR11-12, IA13-16, PR14S, and KS11-12 (Supplementary Table S1). The field experiments were conducted by Kansas State University and Iowa State University (Li *et al.*, 2015, 2018; Mu *et al.*, 2022). The rice population with 174 backcross inbred lines (BILs) from a cross between Koshihikari and Kasalath was evaluated in nine environments, each with a randomized complete block design with two replications. The nine environments were: TS08E, ISA08, TS09, TS07, FU08, TS08L, TH08, HA08, and ISI08. The field experiments were conducted by the National Institute of Agrobiological Sciences Rice Genome Resource Center, Japan (Ma *et al.*, 2002; Onogi *et al.*, 2016), and was used in an earlier study of phenotypic plasticity (Guo *et al.*, 2020).

Flowering time in the sorghum population was recorded as the accumulated growing degree days (GDD) until the day of flowering, and GDD was calculated with the daily maximum and minimum temperatures using 10 °C as the base and 37.8 °C as the maximum. The day of flowering was defined as when 50% of the plants in a plot were at the stage of half of the panicle shedding pollen (Supplementary Table S2). Flowering time in the rice population was the average heading date, recorded for five plants from the middle of the row (Supplementary Table S3).

Genotype data of sorghum RILs included 1462 single nucleotide polymorphisms (SNPs) generated by genotyping by sequencing (Supplementary Table S4). Genotype data of rice BILs included 162 restriction fragment length polymorphisms (RFLPs) (Supplementary Table S5).

### Phenotypic plasticity parameter estimation

The joint regression analysis approach was used to estimate phenotypic plasticity parameters (intercept and slope):


$$\begin{array}{l} Y_{ij}=\mu_{i}+\beta_{i}I_{j}+\delta_{ij} \end{array}$$

where *Y*_*ij*_ is the line mean of the *i*th line in the *j*th environment (*i*=1, 2 … *v*; *j* = 1, 2 … *n*); μ_*i*_ is the mean of the *i*th line over all environments; β_*i*_ is the regression coefficient that measures the response of the *i*th line to environment input; *I*_*j*_ is the environmental mean, expressed as the mean of all lines in the *j*th environment (minus the grand mean so that μ_*i*_ is the intercept); and δ_*ij*_ is the deviation from regression. In the above equation, μ_*i*_ and β_*i*_ are parameters of the intercept and slope, respectively.

We first conducted environment subsetting by extracting a subset of environments from all studied environments. There were 36 subsets when selecting two environments, which is 9 CHOOSE 2, or 9C2. The numbers of subsets for 3–8 environments were 84, 126, 126, 84, 36, and 9, sequentially. For each genotype, 501 pairs of intercepts and slopes were obtained for all these environment subsets. Intercepts and slopes were obtained from the joint regression model for the environment subsets with more than two environments, and for the environment subsets with two environments, averages were used for intercepts and differences for slopes. At the population level, slopes from each subset were compared with the slopes from the whole set of environments for evaluating the consistency of plasticity, and the same was done for intercepts for evaluating the consistency of genotypic mean.

We examined the influence of two factors (environment sample size and environmental mean range) on the change of correlations of phenotypic plasticity parameters between a subset and the whole set. A cube root power function was fitted to the correlation statistics and environmental mean range to better visualize the relationship: *y*=*a*+*bx*^–1/3^+*e* where *y* is correlation of slope; *x* is environmental mean range; *a* and *b* are the coefficients; and *e* is the residual.

### Genomic prediction for phenotypic plasticity

Genomic prediction was conducted for the intercept and slope by using the function mixed.solve in the rrBLUP package (Endelman, 2011). We applied two genomic prediction scenarios for assessing the prediction accuracy of phenotypic plasticity: (A) within a subset of environments and (B) between a subset of environments and the whole set (Supplementary Fig. S2).

(A) The procedure for comparing genomic prediction within a subset of environments was listed as follows (take slope as an example):

1. define a subset of environments;
2. obtain the slopes estimated from the defined subset of environments for all genotypes;
3. choose the fold for cross-validation of genotypes (10-fold was used);
4. predict the slopes of untested genotypes with rrBLUP;
5. calculate the prediction accuracy using the Pearson correlation between the predicted slopes and actual estimated slopes, both under the subset of environments.

(B) The procedure for comparing genomic prediction from the subset of environments with the slope estimated from the whole set was as follows (take slope as an example):

1–4.  same as steps 1–4 in (A);
5. obtain the slopes estimated from the whole set of environments as the actual estimated slopes;
6. calculate the prediction accuracy using the Pearson correlation between the predicted slopes under the subset of environments, and actual estimated slopes across the whole set of environments.

### Modeling the statistics generated from environment subset analysis

Based on the phenotypic plasticity estimation and prediction from 501 subsets and the whole set of environments, we fitted smoothing spline regression models to show the relationship between the environmental mean range and the correlation or prediction accuracy of phenotypic plasticity. Correlation measures the consistency of phenotypic plasticity estimations between the subsets and the whole set of environments. Prediction accuracy measures whether phenotypic plasticity of untested genotypes can be accurately predicted either within a subset of environments or for the whole set of environments. This model fitting was conducted for different environment sample sizes (2–6 environments).

### Searching for the environmental index

The environmental index was detected in previous studies: PTT_18–43_ (photothermal time of 18–43 d after planting) in sorghum (Li *et al.*, 2018) and GDD_9–50_ (growing degree days of 9–50 d after planting) in rice (Guo *et al.*, 2020). These two indices were found to be both biologically relevant and numerically estimable at the growth stage before trait expression to enable trait forecasting for new environments. Weather data used for the environmental index search were collected from NASA POWER (https://power.larc.nasa.gov/) (Supplementary Tables S6, S7).

### Simulation for evaluating environmental variability

To link empirical and simulated experiments for generating additional insights into environmental variability of different testing sites, we applied the same locations, ranges of years, and planting dates in empirical sorghum experiments into simulated experiments, resulting in 1428 environments in sorghum, covering three locations [Iowa (IA), Kansas (KS), and Puerto Rico (PR)], 7 years (2010–2016), and 50 planting dates in summer for all three locations (21 May–10 July) and in winter for PR only (1 December–21 January) (Supplementary Table S8). This same simulation method was applied to rice. As a result, we obtained 1674 environments in six locations over 3 years (2007–2009) and throughout 93 planting dates (30 March–30 June) (Supplementary Table S9). In each crop, nine empirical environments were included in the simulated environments for comparison and interpretation. The planting dates used in simulated environments also considered the actual dates in farmers’ fields (Supplementary Fig. S3). In Kansas, on average, ~30% of fields had been planted in the week of 21 May and almost all fields had been planted in the week of 10 July (data from https://www.nass.usda.gov/). The information for each environment, including coordinate (latitude and longitude), year, and planting date, was used to collect environmental variables, which were then used to obtain the environmental index value (PTT_18–43_ and GDD_9–50_) for each simulated environment. Assuming a constant environmental index was necessary to conduct this simulation analysis about different sites.

The environmental variability in simulations refers to how much the environmental index values differ among trials over years and throughout planting dates. We evaluated the environmental variability for sites of IA, KS, PRS, and PRW in sorghum, where PRS and PRW were considered as two different testing sets due to summer and winter planting resulting in two distinct environmental index distributions. The testing sites ISA, TS, FU, ISI, TH, and HA were all evaluated for environmental variability, but they were formed into four distinct groups by combining TS with FU, and TH with HA because of overlapped distributions and close coordinates of those combined sites.

## Results

### Effects of environment sample size and environmental mean range

**Fig. 2.**
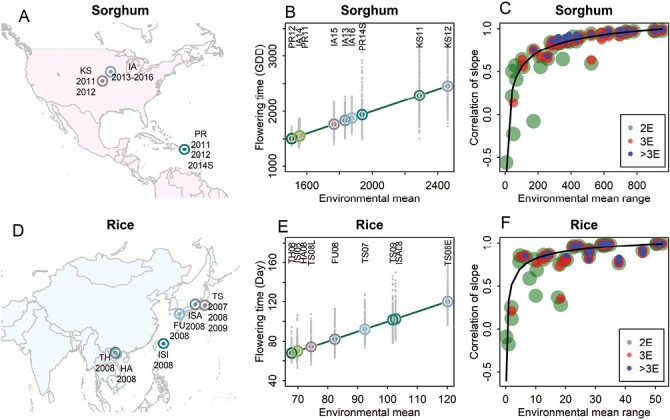
Phenotypic plasticity in flowering time obtained from different subsets of environments. (A) Nine environments for the sorghum population. (B) Phenotypic plasticity in flowering time for the sorghum population. (C) Correlation between slopes estimated from a subset and the whole set of environments for the sorghum population. (D) Nine environments for the rice population. (E) Phenotypic plasticity in flowering time for the rice population. (F) Correlation between slopes estimated from a subset and the whole set of environments for the rice population. In (B) and (E), both environmental mean values (large dots) and trait values of individual genotypes (small gray dots) are plotted. In (C) and (F), each dot represents the correlation of slope between a subset and the whole set; the black line is a fitted curve with a cube root function. 2E, 3E, and >3E denote two, three, and more than three testing environments.

We exploited the flowering time from a sorghum bi-parental population of 237 RILs and from a rice bi-parental population of 174 BILs. The trials of sorghum RILs were conducted in three sites (IA, KS, and PR) and over 6 years (2011–2016), resulting in nine environments (site and year combinations) (Fig. 2A, B; Supplementary Table S1). The sorghum flowering time, quantified in GDD, exhibited a large population-level phenotypic variation across these environments (1507.27 GDD in PR12, ~2459.94 GDD in KS12). The rice trials were conducted in nine environments, covering six sites (TS, FU, ISI, ISA, HA, and TH) and over 3 years (2007–2009). The flowering time mean of the rice BIL population varied across environments, ranging from 63 d in TH08 to 120 d in TS08E (Fig. 2D, E; Supplementary Table S1).

Analysis of phenotypic plasticity often uses the reaction norms of genotypes generated by regressing phenotypes on the environmental mean (average performance of the population of genotypes). This process, termed joint regression analysis, generates a pair of reaction norm parameters for each genotype: intercept (genotypic value) as the expected performance at the average point of the environmental mean values, and slope (plasticity) as the expected degree of performance change in unit change in environmental mean (Finlay and Wilkinson, 1963; Eberhart and Russell, 1966; Li *et al.*, 2018; Guo *et al.*, 2020). We estimated the reaction norm parameters of each genotype for different subsets of environments individually and compared their correlations with the parameters obtained from the whole set of environments. High correlations between slopes were observed when the environmental mean range of a subset covered a large proportion of the whole range (Fig. 2C, F; Supplementary Figs S4–S6). For a subset with a small environmental mean range, the resulting slopes could differ substantially from the overall slopes obtained for the whole set of environments. In extreme cases, negative correlations were observed: –0.56 for slope correlation between the PR12–IA14 pair and the overall for sorghum, and –0.18 for slope correlation between the ISA08–TS09 pair and the overall for rice. Under natural field conditions, while the environmental gradient was dominated by the major environmental factor, some local environmental factors could alter the local reaction norm pattern. When the environmental mean range covered 38% of the whole range in sorghum and 39% in rice, correlations for intercept and slope reached 0.80 or higher. Regarding the environment sample size, high correlations of slopes were obtained for subsets with four or more environments.

Correlation of intercept was also affected by environment sample size and environmental mean range (Supplementary Fig. S7). Overall, the correlations of slope were lower than the correlations of intercept when environments were confined into a small range, reflecting the higher dependence of plasticity (slope) than genotypic value (intercept) on the environmental mean range.

### Genomic prediction of intercept and slope within a subset of environments

**Fig. 3.**
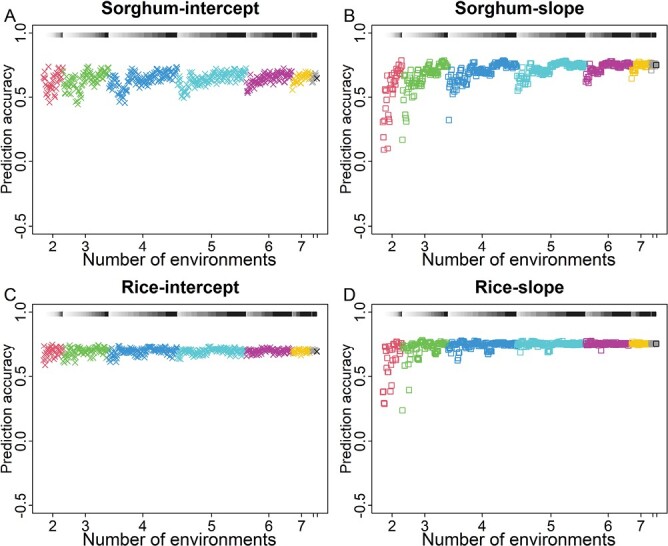
Genomic prediction of intercept and slope within the subsets of environments. (A) Prediction accuracy for intercept in sorghum. (B) Prediction accuracy for slope in sorghum. (C) Prediction accuracy for intercept in rice. (D) Prediction accuracy for slope in rice. Cross-validation within different subsets of environments was conducted for all 501 possible combinations (subsets). The results of the whole set of environments (*n*=9) were plotted for the comparison. The segments with a white to black gradient represent values of environmental mean range, with larger values getting darker.

Next, we checked whether prediction accuracy for genomic prediction of intercept and slope was affected by environment sample size and environmental mean range. Within each subset of environments, we conducted cross-validation, using data from tested genotypes in the training set to generate the genomic prediction model and predict slopes and intercepts of untested genotypes (Supplementary Fig. S2). From two to nine environments for the sorghum population, the minimum prediction accuracy ranged from 0.09 to 0.74 for slope and 0.45 to 0.65 for intercept, and the corresponding values for the rice population were 0.29 to 0.76 for slope and 0.59 to 0.69 for the intercept (Fig. 3). Intercept generally had higher prediction accuracy than slope in experiments with 2–3 environments, but this trend reversed when more environments were involved. These results suggested that environment sample size affected the prediction accuracy of the slope more than that of the intercept.

Environmental mean range also affected prediction accuracy. Both slope and intercept generally showed an increase in prediction accuracy along the gradient of environmental mean range, with the increase being larger for the slope than for the intercept (Fig. 3). For the two-environment cases, slope accuracy increased from 0.09 to 0.78 in sorghum and from 0.29 to 0.77 in rice, and intercept accuracy increased from 0.46 to 0.74 in sorghum and from 0.59 to 0.75 in rice. So, both environment sample size and environmental mean range affected the prediction accuracy of slope more than intercept and, with only a few exceptions, phenotypic plasticity (slope and intercept) of untested genotypes was accurately predicted.

### Genomic prediction of intercept and slope for using a subset of environments

**Fig. 4.**
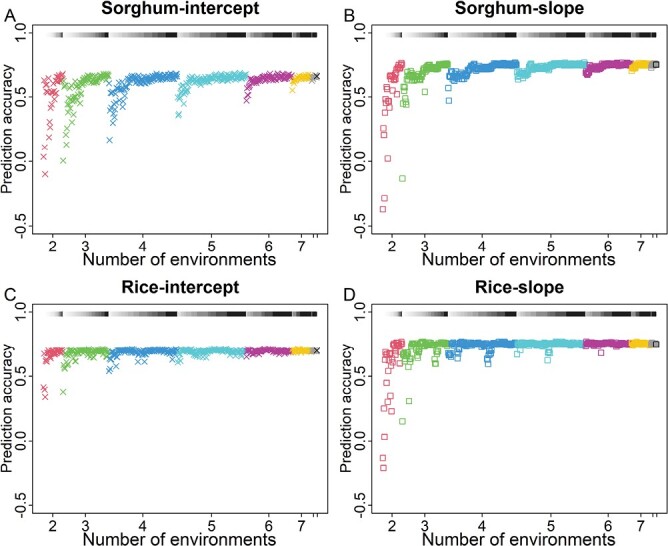
Genomic prediction for phenotypic plasticity across all environments by using a subset of environments. (A) Prediction accuracy for intercept in sorghum. (B) Prediction accuracy for slope in sorghum. (C) Prediction accuracy for intercept in rice. (D) Prediction accuracy for slope in rice. Genomic prediction with different subsets of environments was conducted for all 501 possible combinations (subsets). The results of the whole set of environments (*n*=9) were plotted for the comparison. The segments with a white to black gradient represent values of environmental mean range, with larger values getting darker.

While the previous section checked accuracy of genomic prediction within each defined subset, we were also interested to see how well a training set tested only in a subset of environments can predict the phenotypic plasticity of a validation set across the whole set of environments (Supplementary Fig. S2). For this purpose, a genomic prediction model was established with data of tested genotypes in the subset of environments, and the predicted intercept and slope for untested genotypes were compared with the observed intercept and slope across the whole set of environments. Again, prediction accuracy of both intercept and slope was affected by environment sample size (Fig. 4). As the number of environments increased, the minimum prediction accuracy increased from –0.37 to 0.75 for the slope and from –0.10 to 0.66 for the intercept in sorghum, and from –0.21 to 0.75 for the slope and from 0.34 to 0.70 for the intercept in rice. The prediction accuracy of the intercept and slope was also affected by environmental mean range, and large ranges resulted in high prediction accuracy regardless of environment sample size.

To make it easier to recommend environment sample size and environmental mean range for future experiments, we conducted model fitting and visualized the findings (Supplementary Figs S8, S9). The model fitting used earlier results generated from analyses of environment subsets (Figs 2–4; Supplementary Fig. S7) for curve fitting. The correlation and prediction accuracy, characterizing the phenotypic plasticity consistency between the subsets and the whole set of environments, were modeled with environmental mean range for each environment sample size. The experiments in a small environmental mean range obtained various levels of consistency for slope, being the lowest in two environments and the highest in six environments. With the increase of environmental mean range, curves representing different environment sample sizes converged to the same point, namely the highest consistency level (Supplementary Fig. S8). In other words, a high consistency of slope could be achieved with two resource allocation strategies: choosing environments to obtain a large environmental mean range or increasing environment number. Although intercept had fewer requirements than slope to obtain a high consistency between the subsets and the whole set of environments, a large environmental mean range and more environments were still favored (Supplementary Fig. S9).

### The two most contrasting environments

**Fig. 5.**
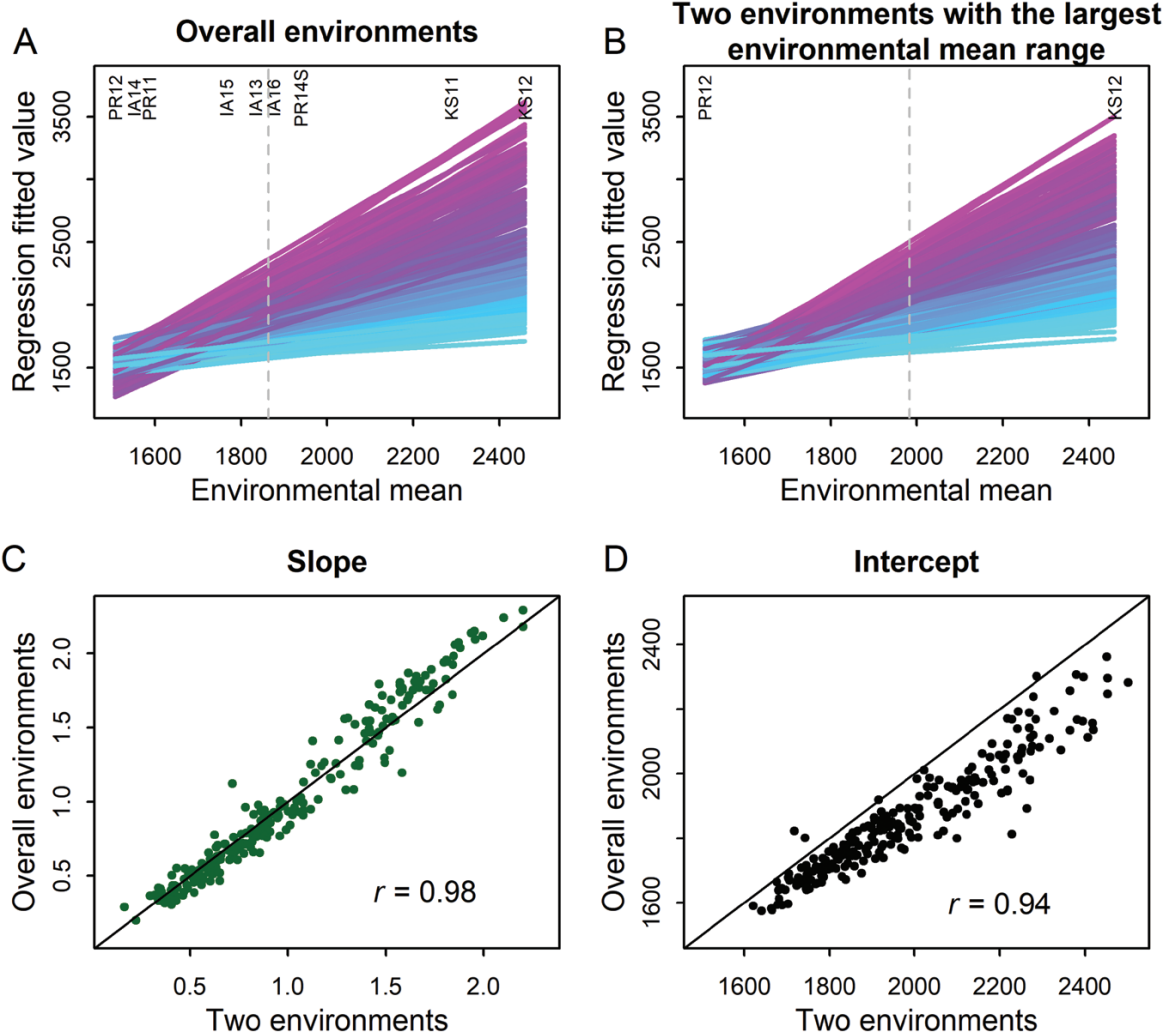
Comparison of phenotypic plasticity between the whole set of nine environments and two extreme environments in sorghum. (A) Reaction norms across the whole set of nine environments. (B) Reaction norms from two environments with the largest environmental mean range. (C and D) Correlation between nine and two environments for slope (C) and intercept (D).

One commonly used strategy to reduce cost in phenotypic plasticity studies was the desire to involve a small number of environments or only two contrasting conditions that would potentially result in a large environmental mean range, which generally covers a large proportion of the entire target range. To relate to this practice, we selected the environments of PR12 and KS12 in sorghum and the environments of TH08 and TS08E in rice for further analyses (Fig. 5). The reaction norms of 237 sorghum genotypes in two environments (PR12 and KS12) were highly similar to those from the whole set of environments, 0.98 for slope correlation and 0.94 for intercept correlation. Parallel results were obtained in rice, with correlation being 0.99 for slope and 0.98 for intercept (Supplementary Fig. S10). High correlation values and very similar parameter values indicated that two extreme environments with a large environmental mean range could capture the overall phenotypic plasticity from the whole set of environments. However, the assumptions in this part of the analysis were that there was a linear reaction norm pattern across the environments and that this pattern was well captured by two extreme environments. This part of the analysis only represents an initial step with examples where these assumptions hold.

### Environmental variability of testing sites via simulation

To provide insights into allocating testing sites to achieve a good coverage of the target population of environments, we simulated 1428 environments (site, year, and planting date combinations) in sorghum and 1674 environments in rice to evaluate the environmental variability of testing sites (Supplementary Table S10). Ideally, the environmental variability refers to how much the environmental mean values would have differed among trials over years and throughout planting dates if all experiments had been conducted. Because all environmental mean values as outcomes of experiments are not possible to obtain in simulations and in actual practice, we used the environmental index, PTT_18–43_ in sorghum and GDD_9–50_ in rice, to measure the environmental variability. The justification was that they were found to be highly correlated with the environmental means obtained from the original experiments and that they can be computed to serve as a surrogate in this assessment. Nevertheless, the assumption that the environmental index detected from those tested environments is the same for all these simulated environments requires future investigations. We made this assumption to enable the environmental variability analysis of testing sites.

We quantified the contributions from the site, year, and planting date to the variance of the environmental index values (Supplementary Table S11). Site and year were significant factors and contributed 67.01% and 11.39% of variance, respectively. Within each site, the year was the main factor, contributing 57.83% and 93.17% of the variance in sorghum and rice, respectively (Supplementary Table S12). We observed that environmental index values in high-latitude sites varied among years, while those in low-latitude sites were similar across years and planting dates (Supplementary Figs S11, S12).

**Fig. 6.**
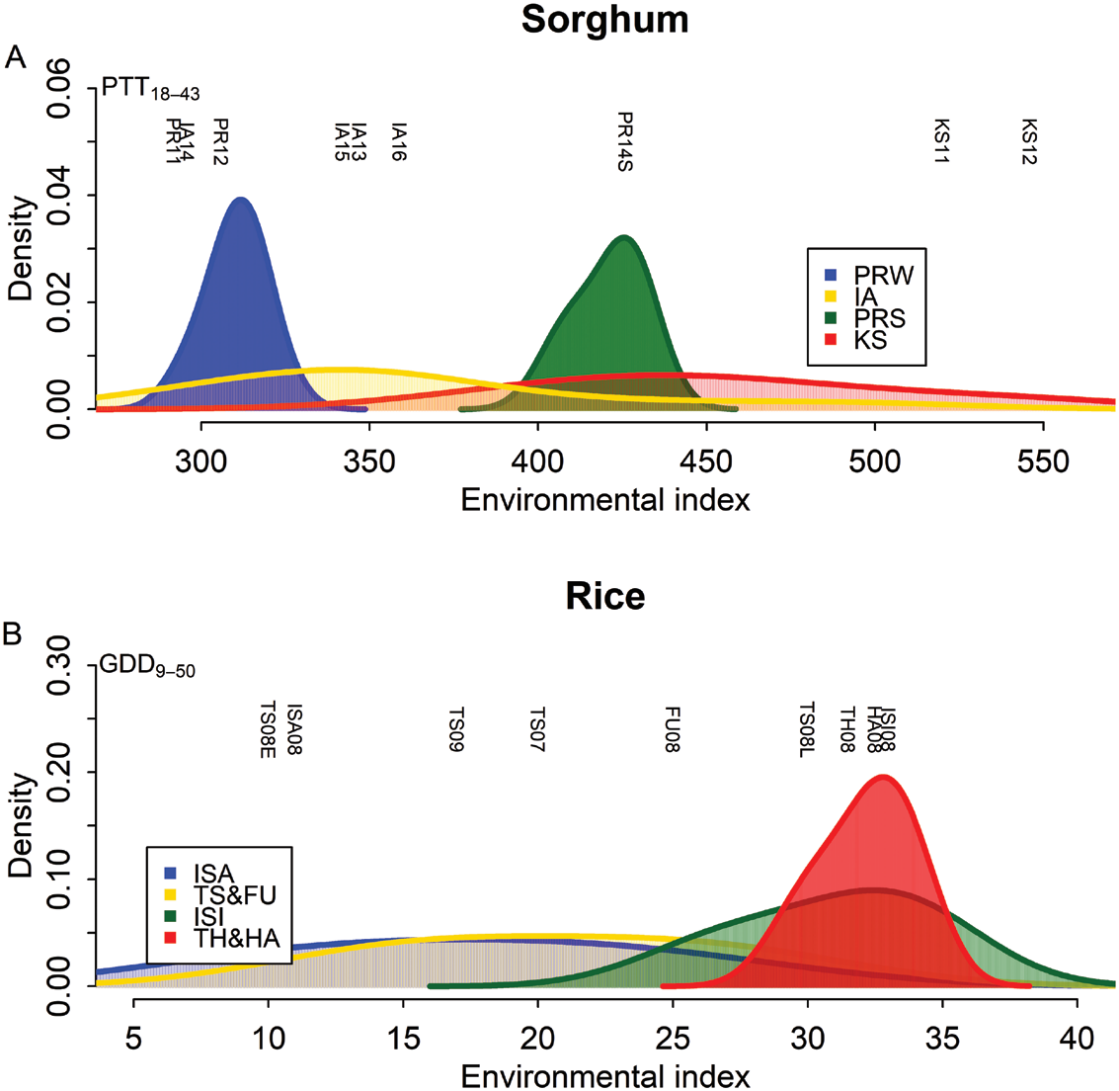
Probability distributions of the environmental index in testing sites. (A) Distribution of the environmental index in sorghum and positions of nine empirical environments. PRS and PRW were considered two different testing sites due to distinct environmental index distributions. (B) Distribution of the environmental index in rice and positions of nine empirical environments. TS and FU were combined, and so were TH and HA because of overlapped distributions and close coordinates of the combined sites.

We plotted the probability distribution of environmental index values for each site (Fig. 6). IA and KS had a much larger environmental variability than PR (PRW in winter and PRS in summer). The distributions of the environmental index overlapped between PRW and IA (e.g. PR11 was close to IA14), and between PRS and KS, suggesting that, for flowering time, similar values of the environmental index can be found in different geographic sites. Interestingly, this distribution explained not only why we originally encountered IA14, which would have been expected to be typical from the distribution, but also that the occurrence of KS11 and KS12, which were not typical from the distribution, allowed us to study the phenotypic plasticity of sorghum flowering time with a large environmental range. In rice, sites of ISA and TS+FU showed large environmental variability, while ISI and TH+HA concentrated on one end of the environmental index. As expected, in both crops, testing sites with large environmental variability were located in high latitudes due to the wider fluctuation in temperature.

## Discussion

Phenotypic plasticity of a given genotype is controlled by genetics and environment (Nicotra *et al.*, 2010). Our work analyzed the effect of tested environments on the estimation and prediction of phenotypic plasticity (Fig. 1; Supplementary Fig. S1). By using genetic populations of sorghum and rice, we examined the consistency of parameter estimation between the subsets and the whole set of environments, and quantified the effects of environment sample size and environmental mean range on the consistency (Fig. 2; Supplementary Figs S4–S7). By using genomic prediction (Supplementary Fig. S2), we found that phenotypic plasticity parameters could be predicted with good accuracy for either within the subset of environments or between the subsets and the whole set, when an adequate environmental mean range or environment sample size was used (Figs 3, 4). By conducting simulations with historical weather data and varying planting dates, we found that the testing sites with large environmental variability should be prioritized for phenotypic plasticity studies (Fig. 6). The general context for the current research is that the reaction norms of the traits generally follow a linear pattern across the range of environments studied (Fig. 1). Future research can work on more complex reaction norm patterns.

Although it is known that phenotypic plasticity can be specific to particular environmental influences (Bradshaw, 1965), whether the major environmental variable remains the dominant pattern when the sample size varies has not been well studied. Allocation of testing sites and planting dates that do not cover the target population of environments well could result in inaccurate parameter estimation of phenotypic plasticity for the tested genotypes. Our study demonstrated that both environment sample size and environmental mean range influenced parameter estimation of phenotypic plasticity from different subsets of environments. While both factors are critical, our results indicated that considerations leading to an increase of environmental mean range should be given priority in study design. Indeed, because increasing environment sample size involves more resources to conduct experiments, it has been a common practice for researchers to choose contrasting treatment or environmental conditions, or a set of conditions optimally spaced within the biologically relevant range to conduct experiments (Des Marais *et al.*, 2012; Sasaki *et al.*, 2015). Our analysis provided quantitative justification for this type of phenotypic plasticity study, which can be under either natural field conditions or controlled conditions (Fig. 5; Supplementary Fig. S10). On the other hand, studies under controlled conditions, with a focus on either individual genes and pathways or genomic prediction, need to be guided and validated under natural field conditions to realize the potential of the discoveries.

Because the environmental index, measured by variables that describe field environments, was thought to be genotype independent and performance free, it has been suggested and examined decades ago (Finlay and Wilkinson, 1963; Eberhart and Russell, 1966; Hardwick and Wood, 1972; Wood, 1976). However, the approach to searching for an environmental index from the combinations of environmental variables and growth windows to replace the environmental mean from performance data was initiated recently (Li *et al.*, 2018; Guo *et al.*, 2020). Once the environmental index was obtained and assumed to be applicable, it could be used to predict trait performance across different environments and to provide an explicit environmental gradient to show reaction norms at allele, gene, haplotype, and individual levels (Li *et al.*, 2018, 2021; Millet *et al.*, 2019; Guo *et al.*, 2020; Mu *et al.*, 2022). On the other hand, such an environmental index and the observed environmental mean (itself being an index) are context dependent, meaning that the environmental variables and associated windows identified from the tested environments need to remain relevant for new environments. This reasoning agrees with what we observed from the analyses when the entire data were subsetted to estimate phenotypic plasticity. When the environmental mean range or environment sample size is small, local environmental variables may affect the plant growth and development to a higher degree such that the resulting phenotypic plasticity deviates more from what is expected from the overall pattern driven by the global environmental variables across the whole range (Fig. 1). Whether the focus of a study is the entire growing range of a species or only a particular ecological niche needs to be defined before the environmental sampling and analyses to identify the environmental index and to obtain reaction norms are carried out. Nevertheless, additional insights gained from physiological studies and crop growth models in the same or related species (Technow *et al.*, 2015; Bustos-Korts *et al.*, 2019) are helpful in guiding this process of searching for the environment index and using it for performance prediction and genetic dissection.

It is necessary to explicitly point out the assumptions in the current study. First, the findings of the current study are conditioned on the assumption of a linear reaction norm pattern across the environments. Second, by using the parameters obtained from the whole set of environments as the standard for comparison, we assumed that the maximum possible and biologically permissible environmental range was of primary interest and that the major environmental factor and growth window driving plasticity across this range were therefore of the most importance. In other words, we evaluated how well different subsets of environments approximated the results of the whole set. Under the first two assumptions, the recommendation from our analysis is to conduct experiments in environments that are more likely to either generate a large environmental mean range or increase the environment sample size. Third, to examine the environmental variability of testing sites, we had to assume that the environmental index remained the same for the simulated environments as that identified from the tested environments. Otherwise, we would not be able to conduct the analysis before a very large number of experiments were carried out at those testing sites by varying year and planting date. With the third assumption and turning attention to testing sites, it appears that the most economical strategy is to select sites that can generate the largest and most probable environmental mean difference, particularly at the early stage of the research before many more environments are investigated. Future research may further revise these recommendations.

With new envirotyping technologies (Xu, 2016; Cooper and Messina, 2021; Crossa *et al.*, 2021; Resende *et al.*, 2021), enviromics can be conducted for understanding environmental heterogeneity and its impact on plant growth and development. Built on the earlier success of the establishment of models for optimizing trial allocation to subregions (González-Barrios *et al.*, 2019; Prus and Piepho, 2021), more considerations from envirotyping can incorporate information from climate, soil, and cropping system. Extensive phenotyping throughout the season can also help. On the grand scale, this aspect of research contributes to the overall design of crop breeding to leverage genomics and allied technologies and strategies (Varshney *et al.*, 2021; Xu *et al.*, 2022), to gain a better understanding of genetic architecture of complex traits, mine genebank accessions for superior alleles, and breed crops with climate resiliency.

Improved designs of CGEs and METs are important to gain mechanistic insights into genetics and environments and to facilitate breeding programs to develop cultivars with different adaptation zones. With a systematic quantitative assessment under the phenotypic plasticity framework, the current study provided guidance for planning of future CGEs and METs, as well as for connecting molecular and physiological studies under controlled conditions or natural field conditions. Our simulation analysis of the environmental index generated additional insights into how to leverage environmental variability for testing site allocation. Continued integration of genomics, enviromics, physiology, and analytics would further enhance our ability to explain, to predict, to act, and to adapt.

## Supplementary data

The following supplementary data are available at *JXB* online.

Fig. S1. The design of this study to investigate the consistency of phenotypic plasticity estimation.

Fig. S2. Genomic prediction of phenotypic plasticity.

Fig. S3. Sorghum planting dates in Kansas across 12 years.

Fig. S4. Reaction norms of subsets of environments with small, medium, and large environmental index ranges.

Fig. S5. Correlations between the whole set and subsets of two environments for slope and intercept in sorghum.

Fig. S6. Correlations between the whole set and subsets of two environments for slope and intercept in rice.

Fig. S7. Effects of environment sample size and environmental mean range on correlations of flowering time phenotypic plasticity between subsets and the whole set.

Fig. S8. Fitted lines of slope estimations and predictions for recommending environment sample size and environmental mean range.

Fig. S9. Fitted lines of intercept estimations and predictions for recommending environment sample size and environmental mean range.

Fig. S10. Comparison of phenotypic plasticity estimates between the whole set of nine environments and two contrasting environments in rice.

Fig. S11. Changes in environmental index values across years and planting dates in testing sites in Iowa, Kansas, and Puerto Rico.

Fig. S12. Changes in environmental index values across years and planting dates in TS, FU, ISA, ISI, TH, and HA.

Table S1. Description of the nine environments used for multi-environment trials in sorghum and rice.

Table S2. Flowering time measured as the accumulated growing degree days in nine environments each having two replications in sorghum.

Table S3. Flowering time measured as the days after planting in nine environments each having two replications in rice.

Table S4. Genotypic information of 1426 SNPs for 237 recombinant inbred lines.

Table S5. Genotypic information of 162 restriction fragment length polymorphic markers for 176 backcross inbred lines.

Table S6. Weather data in the empirical multi-environment trials in sorghum.

Table S7. Weather data in the empirical multi-environment trials in rice.

Table S8. Weather data in the simulation study in sorghum.

Table S9. Weather data in the simulation study in rice.

Table S10. Description of empirical and simulated experiments across years and planting dates.

Table S11. Variance partitioning of the environmental index into site, year, planting date, and residuals in the simulated experiments.

Table S12. Variance partitioning of the environmental index within each testing site into year, planting date, and residuals.

erad398_suppl_Supplementary_Figures_S1-S12_Tables_S1_S10-S12

erad398_suppl_Supplementary_Tables_S2-S9

## Data Availability

All data are included in the manuscript and its supplementary data. Data for phenotype, genotype, and weather information are provided in Supplementary Tables S2–S7. Data for weather information in simulations are provided in Supplementary Tables S8 and S9.
