# Supplementary material for: Environmental context of phenotypic plasticity in flowering time in sorghum and rice
Source: J Exp Bot. 2023 Oct 11;75(3):1004–15. doi: 10.1093/jxb/erad398 (PMC10837014; doi:10.1093/jxb/erad398)
Supplement: erad398_suppl_Supplementary_Figures_S1-S12_Tables_S1_S10-S12 [file erad398_suppl_supplementary_figures_s1-s12_tables_s1_s10-s12.pdf]

Environmental Context of Phenotypic Plasticity in Flowering Time in Sorghum and Rice

Tingting Guo[1,2,*], Jialu Wei[3], Xianran Li[4], and Jianming Yu[3,*]

[1] Hubei Hongshan Laboratory, Wuhan, Hubei, China.
[2] College of Plant Science and Technology, Huazhong Agricultural University, Wuhan, Hubei, China.
[3] Department of Agronomy, Iowa State University, Ames, IA, USA.
[4] USDA, Agricultural Research Service, Wheat Health, Genetics, and Quality Research Unit, Pullman, WA, USA.

*Corresponding authors
Tingting Guo, tguo@mail.hzau.edu.cn; and Jianming Yu, jmyu@iastate.edu

The following Supplementary Data is available for this article:
Supplementary Figure S1 – S12
Supplementary Table S1 – S12

Figure S1. The design of this study to investigate the consistency of phenotypic plasticity estimation.
Figure S2. Genomic prediction of phenotypic plasticity.
Figure S3. Sorghum planting dates in Kansas across 12 years.
Figure S4. Reaction norms of subsets of environments with small, medium, and large environmental index ranges.
Figure S5. Correlations between the whole set and subsets of two environments for slope and intercept in sorghum.
Figure S6. Correlations between the whole set and subsets of two environments for slope and intercept in rice.
Figure S7. Effects of environment sample size and environmental mean range on correlations of flowering time phenotypic plasticity between subsets and the whole set.
Figure S8. Fitted lines of slope estimations and predictions for recommending environment sample size and environmental mean range.
Figure S9. Fitted lines of intercept estimations and predictions for recommending environment sample size and environmental mean range.
Figure S10. Comparison of phenotypic plasticity estimates between the whole set of nine environments and two contrasting environments in rice.
Figure S11. Changes in environmental index values across years and planting dates in testing sites in Iowa, Kansas, and Puerto Rico.
Figure S12. Changes in environmental index values across years and planting dates in TS, FU, ISA, ISI, TH, and HA.

Table S1. Description of the nine environments used for multi-environment trials in sorghum and rice.

Table S2. Flowering time measured as the accumulated growing degree days in nine environments each having two replications in sorghum (separate file).
Table S3. Flowering time measured as the days after planting in nine environments each having two replications in rice (separate file).
Table S4. Genotypic information of 1426 SNPs for 237 recombinant inbred lines (separate file).
Table S5. Genotypic information of 162 restriction fragment length polymorphic markers for 176 backcross inbred lines (separate file).
Table S6. Weather data in the empirical multi-environment trials in sorghum (separate file).
Table S7. Weather data in the empirical multi-environment trials in rice (separate file).
Table S8. Weather data in the simulation study in sorghum (separate file).
Table S9. Weather data in the simulation study in rice (separate file).
Table S10. Description of empirical and simulated experiments across years and planting dates.
Table S11. Variance partitioning of environmental index into site, year, planting date, and residuals in the simulated experiments.
Table S12. Variance partitioning of environmental index within each testing site into year, planting date, and residuals.

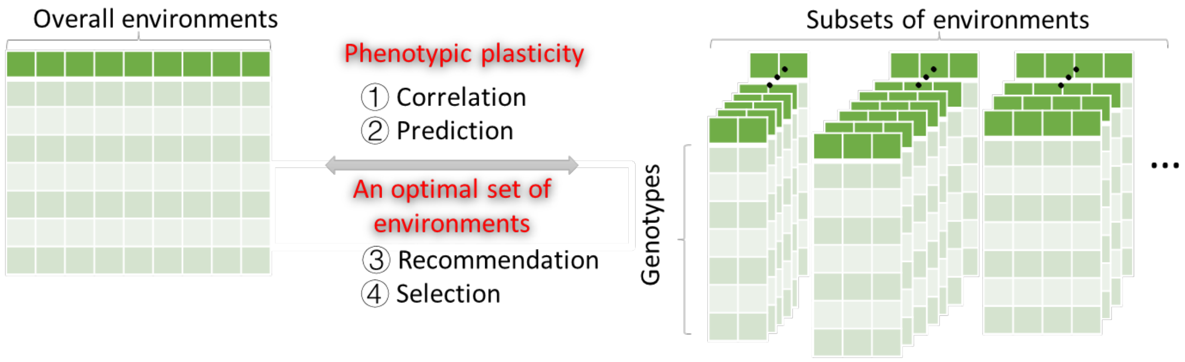

Figure S1. The design of this study to investigate the consistency of phenotypic plasticity estimation. Phenotypic plasticity parameters (intercept and slope) are obtained from the original trait values. The estimated parameter values are compared between subsets and the whole set of environments using correlation and prediction methods. These comparisons provide guidance for selection and recommendation of an optimal set of environments for studying phenotypic plasticity.

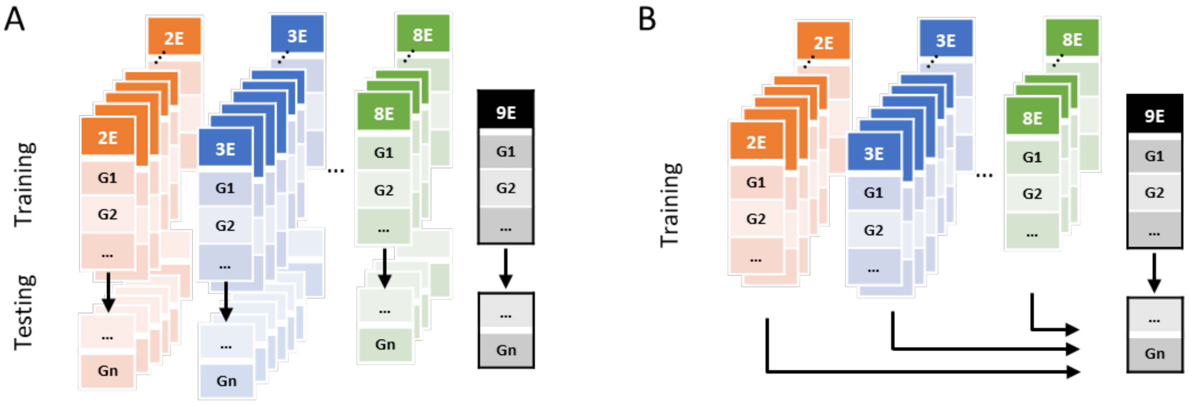

Figure S2. Genomic prediction of phenotypic plasticity. (A) Scenario A: genomic prediction of phenotypic plasticity is performed within subsets of environments. (B) Scenario B: genomic prediction of phenotypic plasticity from subsets of environments is compared with estimates obtained from the whole set.

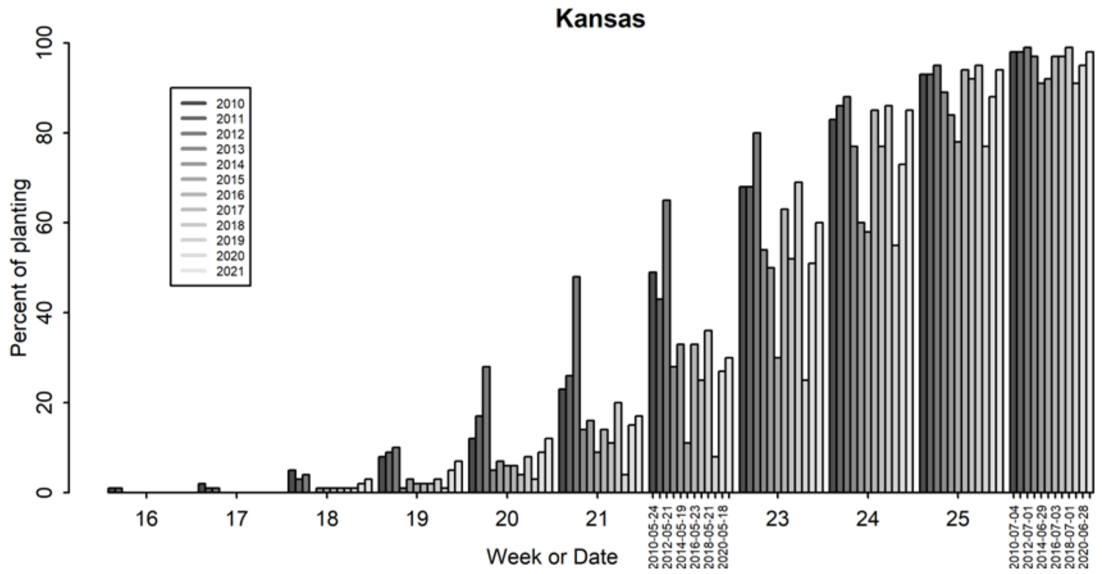

Figure S3. Sorghum planting dates in Kansas across 12 years. On average, about 30% fields are planted in the 22nd week and almost all fields are planted in the 26th week of the year (Data from https://www.nass.usda.gov/) (Using Week as the x-axis).

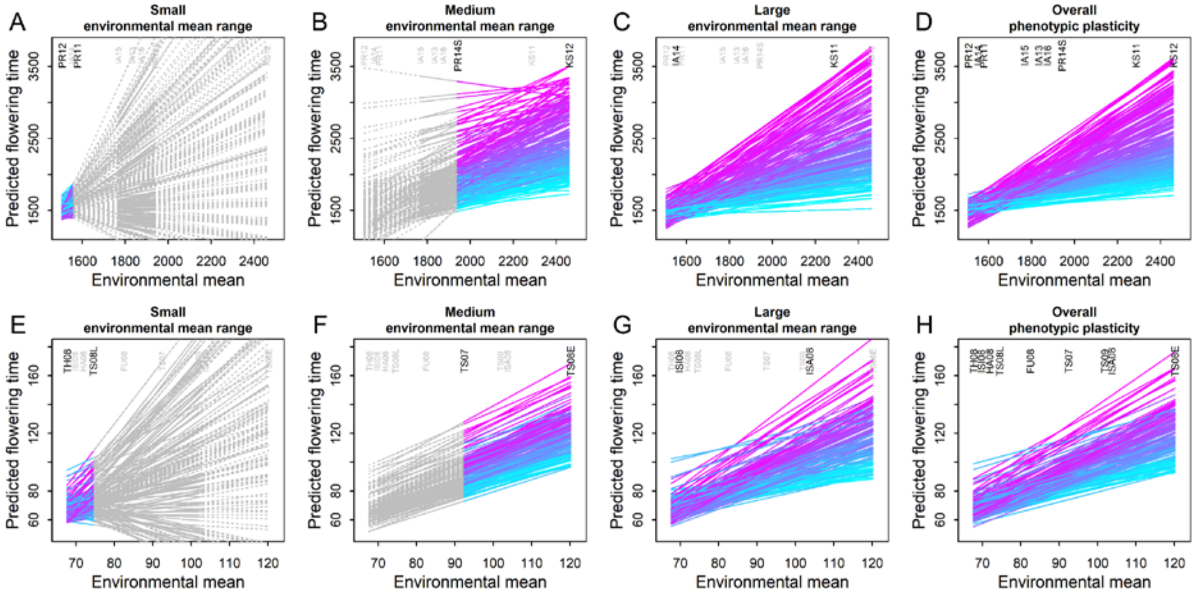

Figure S4. Reaction norms of subsets of environments with small, medium, and large environmental mean ranges. (A-D) Reaction norms of subsets with different ranges and the whole set of environments in sorghum. (E-H) Reaction norms of subsets with different ranges and the whole set of environments in rice.

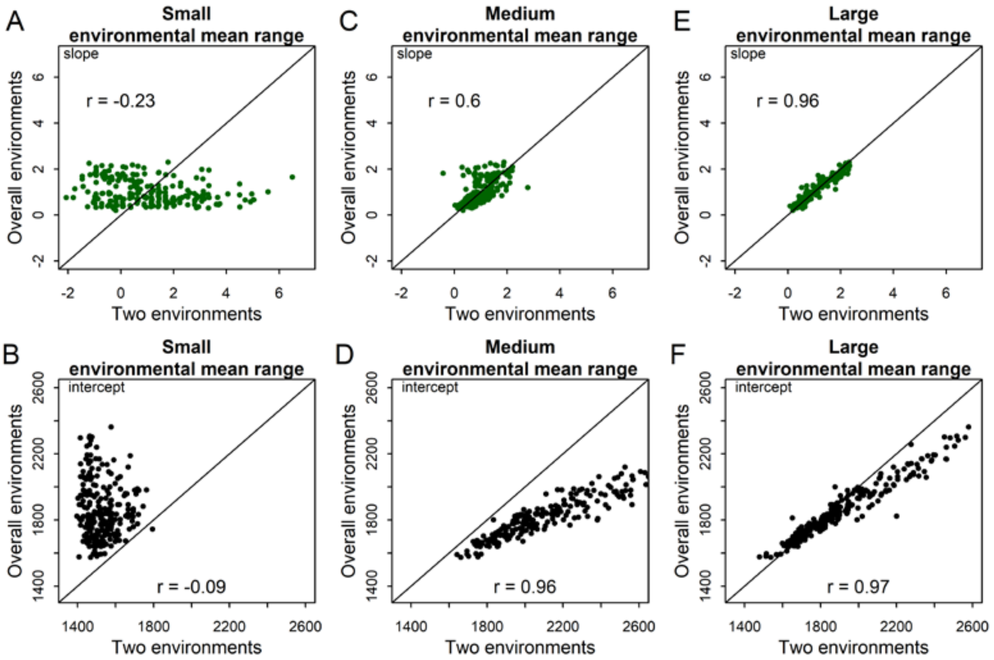

Figure S5. Correlations between the whole set and subsets of two environments for slope and intercept in sorghum. The subsets of two environments with small (A-B), medium (C-D), and large (E-F) environmental mean ranges result in different correlations of slope and intercept.

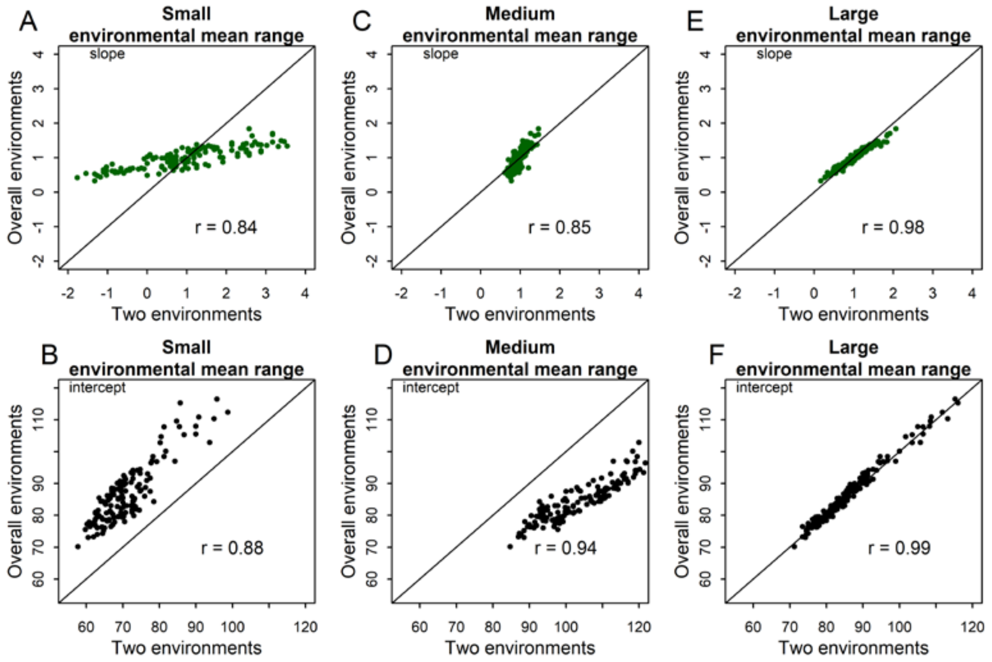

Figure S6. Correlations between the whole set and subsets of two environments for slope and intercept in rice. The subsets of two environments with small (A-B), medium (C-D), and large (E-F) environmental mean ranges result in different correlations of slope and intercept.

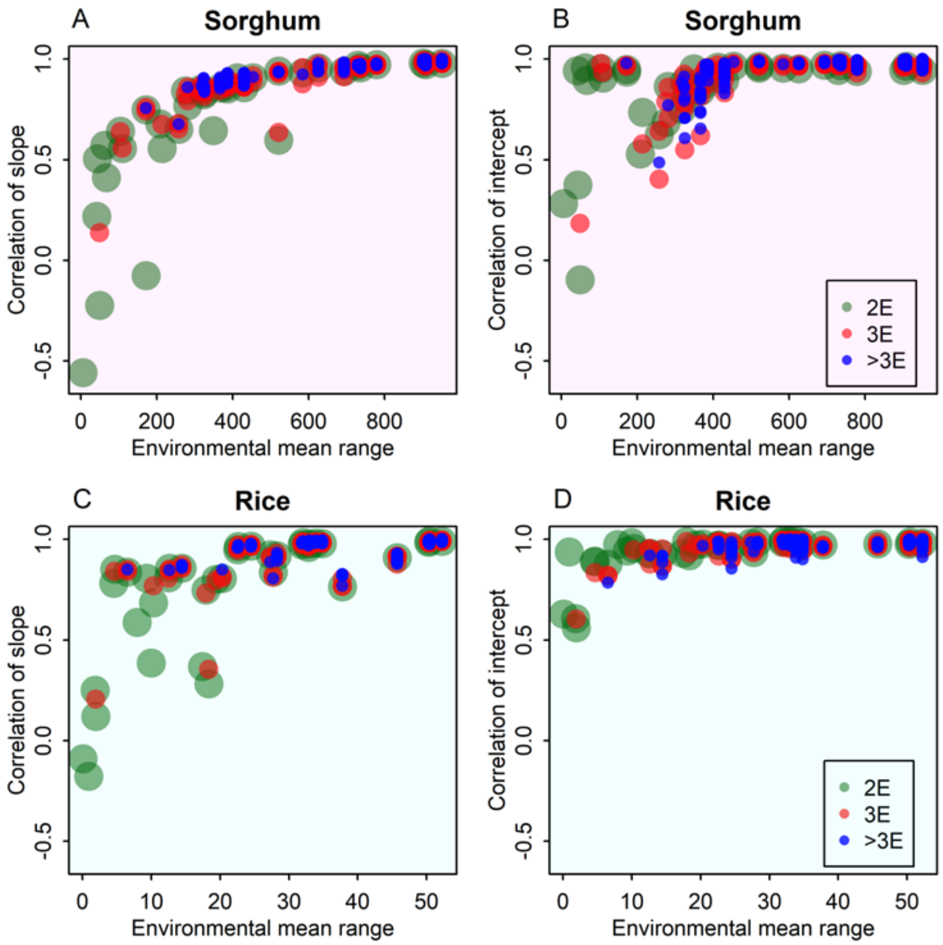

Figure S7. Effects of environment sample size and environmental mean range on correlations of flowering time phenotypic plasticity between subsets and the whole set. The subsets of environments are categorized into three groups: two environments or 2E, three environments or 3E, and more than three environments (>3E). (A, C) Correlations between subsets and the whole set for slopes in sorghum (A) and rice (C). (B, D) Correlations between subsets and the whole set for intercepts in sorghum (B) and rice (D).

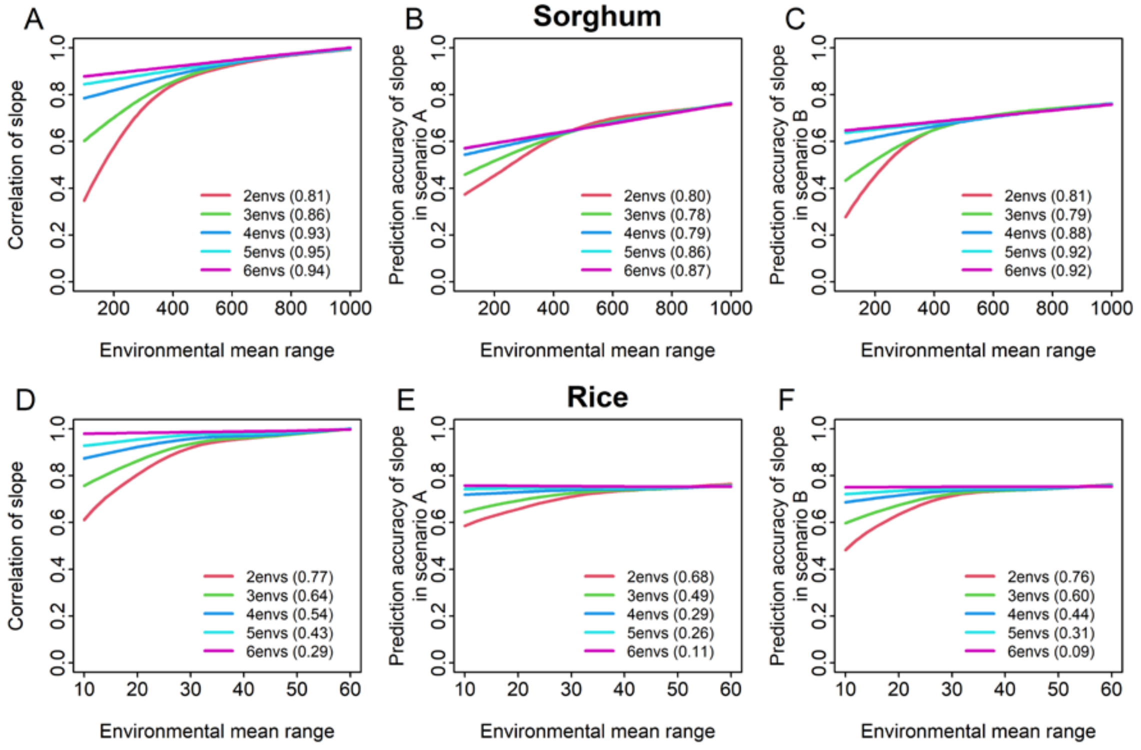

Figure S8. Fitted lines of slope estimations and predictions for recommending environment sample size and environmental mean range. (A, D) Correlations between subsets and the whole set of environments for slopes in sorghum (A) and rice (D). (B, E) Genomic prediction accuracy for slopes of untested genotypes within subsets of environments in sorghum (B) and rice (E) (Scenario A). (C, F) Genomic prediction accuracy for slopes of untested genotypes across the whole range in sorghum (C) and rice (F) (Scenario B). Scenario A and Scenario B are shown in **Figure S2**. The number in the parentheses represents how well the regression model fits the data.

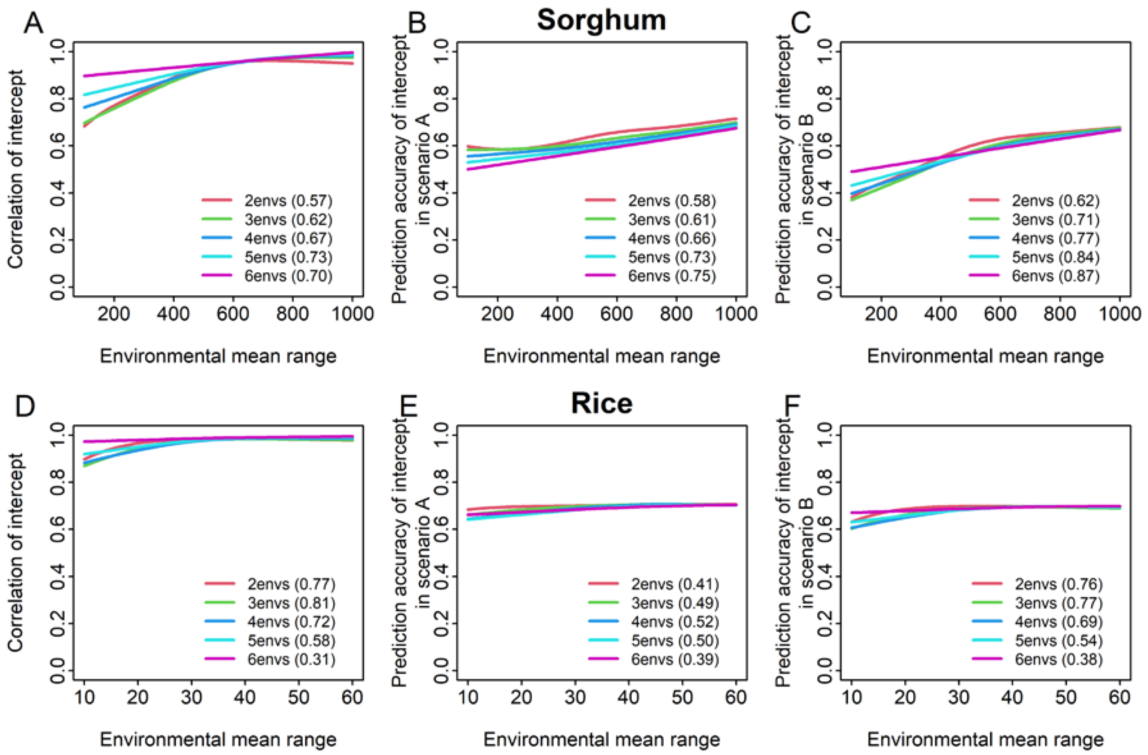

Figure S9. Fitted lines of intercept estimations and predictions for recommending environment sample size and environmental mean range. (A, D) Correlations between subsets and the whole set of environments for intercepts in sorghum (A) and rice (D). (B, E) Genomic prediction accuracy for intercepts of untested genotypes within subsets of environments in sorghum (B) and rice (E) (Scenario A). (C, F) Genomic prediction accuracy for intercepts of untested genotypes across the whole range in sorghum (C) and rice (F) (Scenario B). Scenario A and Scenario B are shown in **Figure S2**. The number in the parentheses represents how well the regression model fits the data.

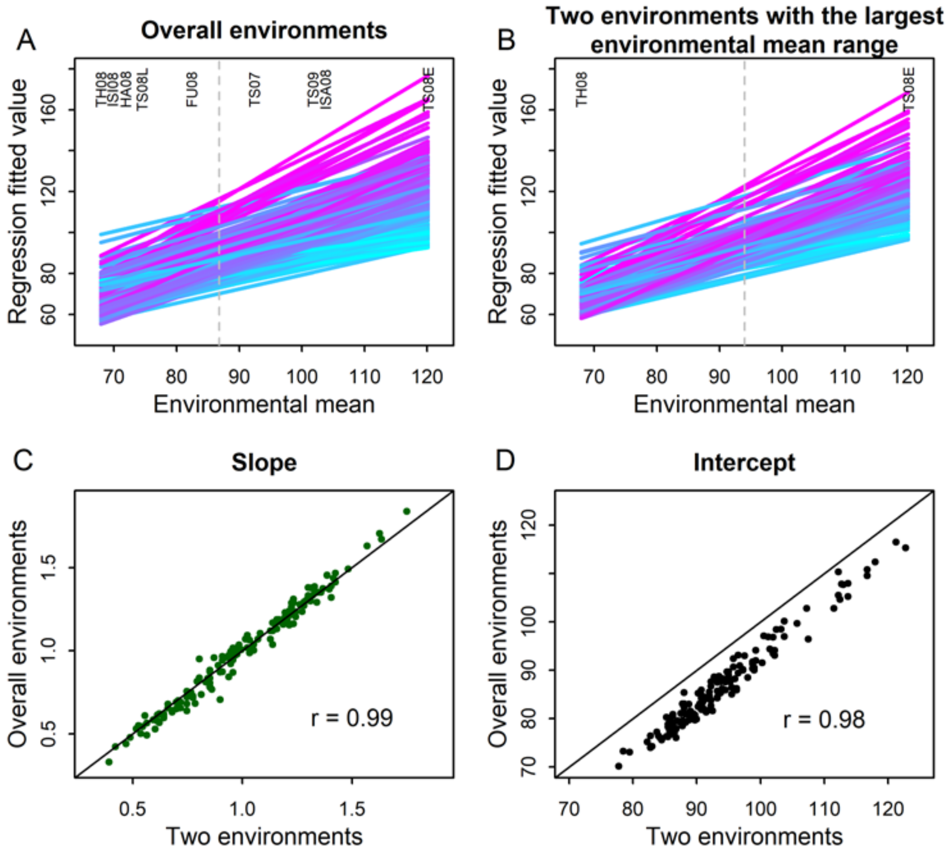

Figure S10. Comparison of phenotypic plasticity estimates between the whole set of nine environments and two contrasting environments in rice. (A) Reaction norms across nine environments. (B) Reaction norms from two environments with the largest environmental mean range. (C-D) Correlation between nine and two environments for slope (C) and intercept (D).

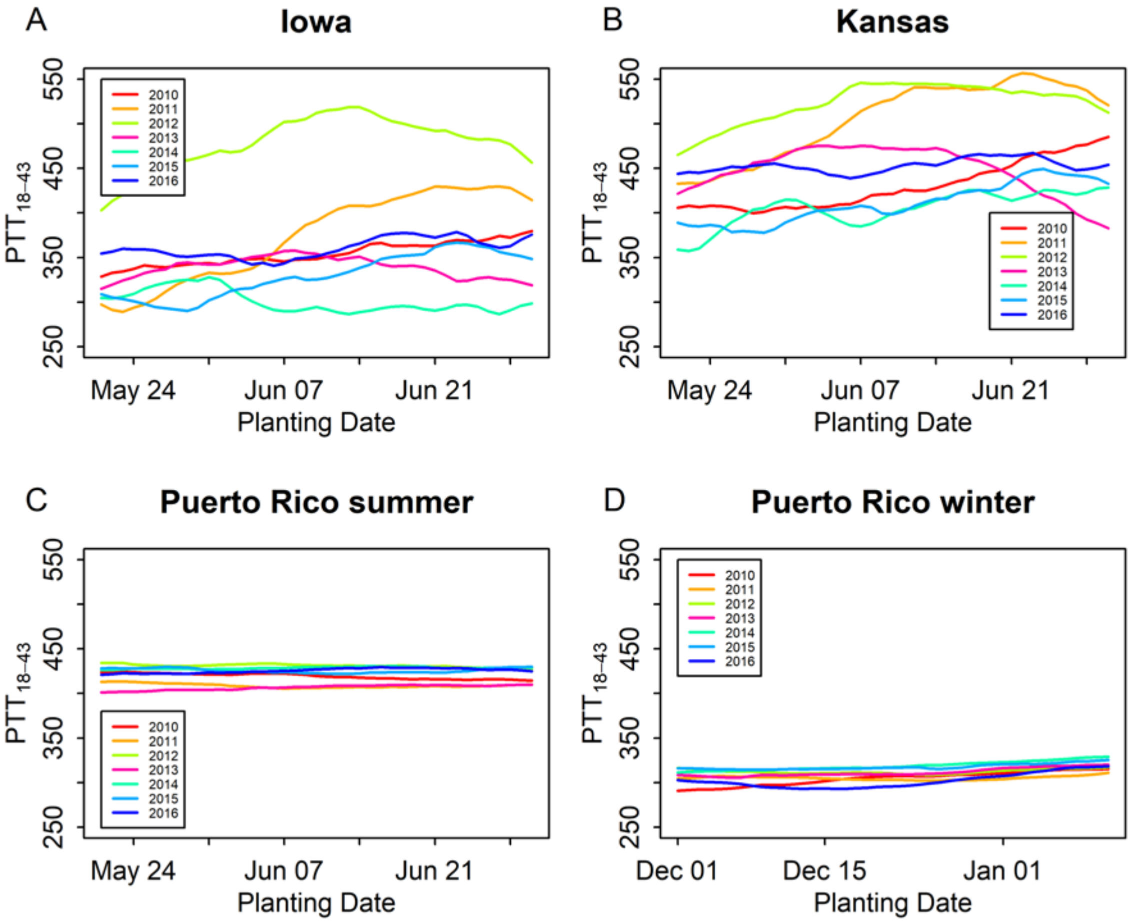

Figure S11. Changes in environmental index values across years and planting dates in testing sites in Iowa, Kansas, and Puerto Rico. (A-B) Variability of the environmental index $PTT_{18\text{-}43}$ (photothermal time from 18-43 days after planting) observed in Iowa (A) and Kansas (B) across different years and planting dates. (C-D) Consistency of the environmental index $PTT_{18\text{-}43}$ observed in Puerto Rico during both summer (C) and winter (D), regardless of the year or planting date.

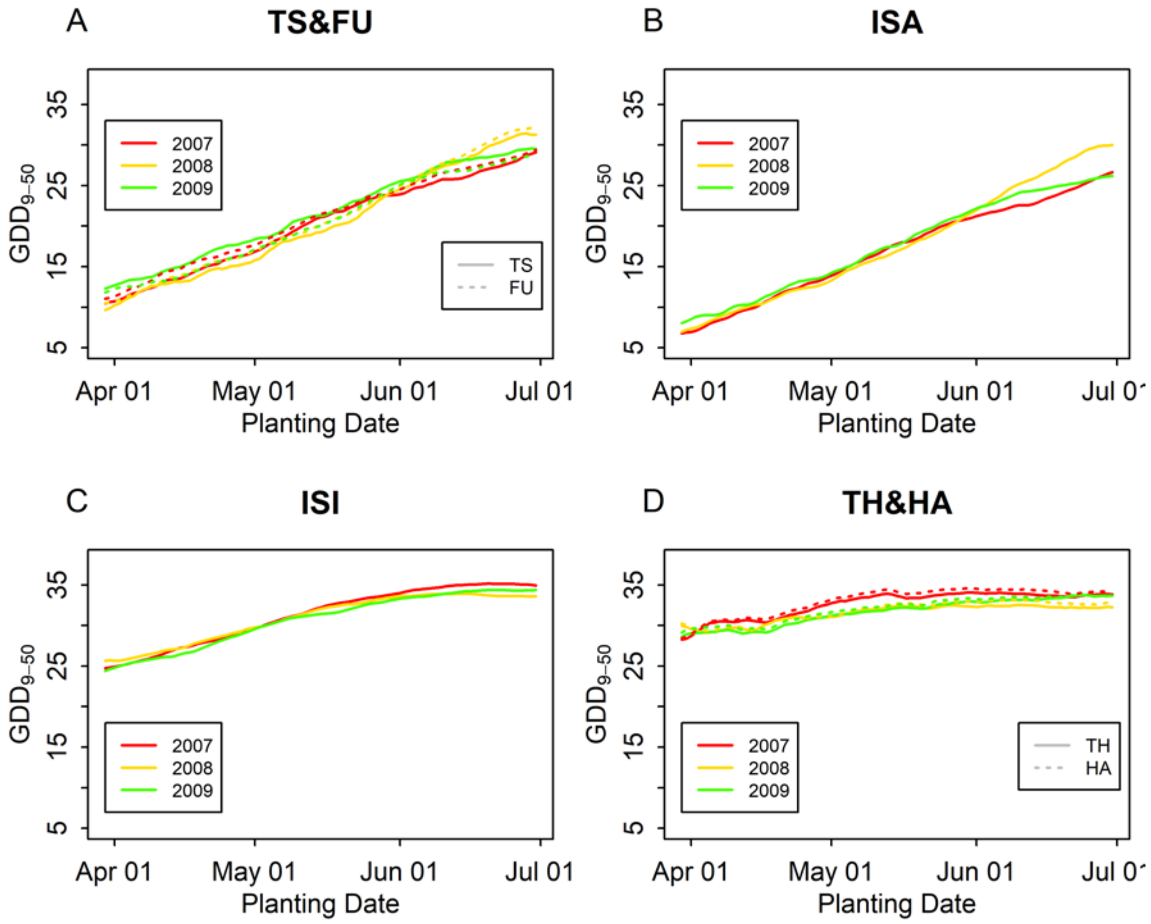

Figure S12. Changes in environmental index values across years and planting dates in TS, FU, ISA, ISI, TH, and HA. (A-B) Variability of the environmental index GDD_{9-50} (Growing degree days from 9-50 days after planting) observed in TS&FU and ISA, with greater variation across planting dates but less variation across years. (C) The environmental index GDD_{9-50} in ISI shows an increase with delayed plantings but remains consistent across years. (D) The environmental index GDD_{9-50} shows slight variation among years and planting dates in TH&HA. TS and FU sites are combined, as well as TH and HA sites, due to overlapping distributions and close coordinates.

Table S1. Description of the nine environments used for multi-environment trials in sorghum and rice.

Experiment	Env_code[a]	Latitude	Longitude	PlantingDate	TrialYear	Location
Sorghum	PR11	18.04	-66.80	12/4/2010	2010	Puerto Rico
	PR12	18.04	-66.80	12/12/2011	2011	Puerto Rico
	KS11	39.18	-96.57	6/8/2011	2011	Kansas
	KS12	39.18	-96.57	6/7/2012	2012	Kansas
	IA13	42.03	-93.63	6/3/2013	2013	Iowa
	IA14	42.03	-93.63	6/10/2014	2014	Iowa
	PR14S	18.04	-66.80	6/5/2014	2014	Puerto Rico
	IA15	42.03	-93.63	6/17/2015	2015	Iowa
	IA16	42.03	-93.63	5/23/2016	2016	Iowa
Rice	TS07	36.01	140.06	5/14/2007	2007	Tsukuba
	TS08E	36.01	140.06	3/31/2008	2008	Tsukuba
	TS08L	36.01	140.06	6/23/2008	2008	Tsukuba
	TS09	36.01	140.06	4/28/2009	2009	Tsukuba
	ISA08	36.3	136.36	4/22/2008	2008	Ishikawa
	FU08	33.37	130.28	6/3/2008	2008	Fukuoka
	ISI08	24.23	124.11	6/25/2008	2008	Ishigaki
	TH08	21.35	105.49	6/30/2008	2008	Thai Nguyen
	HA08	21.01	105.8	6/16/2008	2008	Ha Noi

Note: [a], the environment name, a combination of location and trial year (and early (E), late (L), or summer (S) planting).

Table S2. Flowering time measured as the accumulated growing degree days in nine environments each having two replications in sorghum (separate file).

Table S3. Flowering time measured as the days after planting in nine environments each having two replications in rice (separate file).

Table S4. Genotypic information of 1426 SNPs for 237 recombinant inbred lines (separate file).

Table S5. Genotypic information of 162 restriction fragment length polymorphic markers for 176 backcross inbred lines (separate file).

Table S6. Weather data in the empirical multi-environment trials in sorghum (separate file).

Table S7. Weather data in the empirical multi-environment trials in rice (separate file).

Table S8. Weather data in the simulation study in sorghum (separate file).

Table S9. Weather data in the simulation study in rice (separate file).

Table S10. Description of empirical and simulated experiments across years and planting dates.

Experiment	Location	Latitude	Longitude	TrialYear	PlantingDate
Sorghum Observation	IA	42.0308	-93.6319	2013-2016	06/03, 06/10, 06/17, 05/23
	KS	39.1836	-96.5717	2011-2012	06/08, 06/07
	PRS	18.0373	-66.7963	2014	06/05
	PRW	18.0373	-66.7963	2011-2012	12/04, 12/12
Sorghum Simulation	IA	42.0308	-93.6319	2010-2016	05/21-07/10
	KS	39.1836	-96.5717	2010-2016	05/21-07/10
	PRS	18.0373	-66.7963	2010-2016	05/21-07/10
	PRW	18.0373	-66.7963	2010-2017	12/01-01/20
Rice Observation	TS	36.01	140.06	2007-2009	03/31, 04/28, 05/14, 06/23
	ISA	36.3	136.36	2008	04/22
	FU	33.37	130.28	2008	06/03
	ISI	24.23	124.11	2008	06/25
	TH	21.35	105.49	2008	06/30
	HA	21.01	105.8	2008	06/16
Rice Simulation	TS	36.01	140.06	2007-2009	03/30-06/30
	ISA	36.3	136.36	2007-2009	03/30-06/30
	FU	33.37	130.28	2007-2009	03/30-06/30
	ISI	24.23	124.11	2007-2009	03/30-06/30
	TH	21.35	105.49	2007-2009	03/30-06/30
	HA	21.01	105.8	2007-2009	03/30-06/30

Table S11. Variance partitioning of environmental index into site, year, planting date, and residuals in the simulated experiments.

Species	Terms	Df	Sum Sq	Mean Sq	F value	Pr(>F)	Percent of Total
Sorghum	Site	3	3E+06	1155192	1052.89	<2e-16***	67.01
	Year	6	589057	98176	89.482	<2e-16***	11.39
	Year/Planting date	280	175454	627	0.571	1	3.39
	Residual	858	941362	1097			18.20
Rice	Site	5	63233	12647	2107.75	<2e-16***	64.52
	Year	2	24	12	2.006	2.006	0.02
	Year/Planting date	276	26410	96	15.948	<2e-16***	26.95
	Residual	1390	8340	6			8.51

Table S12. Variance partitioning of environmental index within each testing site into year, planting date, and residuals.

Species	Site	Terms	Df	Sum Sq	Mean Sq	F value	Pr(>F)	Percent of Total
Sorghum	IA	Year	6	781479	130247	262.53	<2e-16***	80.85
		Planting date	40	66036	1651	3.328	5.17e-09***	6.83
		Residual	240	119069	496			12.32
	KS	Year	6	506960	84493	190.345	<2e-16***	72.81
		Planting date	40	82793	2070	4.663	2.38e-14***	11.89
		Residual	240	106535	444			15.30
	PRS	Year	6	22843	3807	556	<2e-16***	93.17
		Planting date	40	31	1	0.112	1	0.13
		Residual	240	1643	7			6.70
	PRW	Year	6	10689	1781.5	149.9	<2e-16***	57.83
		Planting date	40	4942	123.6	10.4	<2e-16***	26.74
		Residual	240	2852	11.9			15.43
Rice	TSU	Year	2	95	47.61	65.47	<2e-16***	0.97
		Planting date	92	9608	104.44	143.64	<2e-16***	97.67
		Residual	184	134	0.73			1.36
	ISA	Year	2	35	17.54	20.28	1.10E-08	0.32
		Planting date	92	10783	117.21	135.55	<2e-16***	98.23
		Residual	184	159	0.86			1.45
	FUK	Year	2	13	6.59	9.713	9.77E-05	0.13
		Planting date	92	9569	104.01	153.279	<2e-16***	98.58
		Residual	184	125	0.68			1.29
	ISI	Year	2	14.8	7.391	55.62	<2e-16***	0.52
		Planting date	92	2826.5	30.723	231.19	<2e-16***	98.63
		Residual	184	24.5	0.133			0.85
	THA	Year	2	73.2	36.62	175.1	<2e-16***	10.89
		Planting date	92	560.4	6.09	29.12	<2e-16***	83.38
		Residual	184	38.5	0.21			5.73
	HAA	Year	2	86.8	43.41	283.71	<2e-16***	12.13
		Planting date	92	600.3	6.53	42.65	<2e-16***	83.92
		Residual	184	28.2	0.15			3.94
